# From tools to threats: a reflection on the impact of artificial-intelligence chatbots on cognitive health

**DOI:** 10.3389/fpsyg.2024.1259845

**Published:** 2024-04-02

**Authors:** Ismail Dergaa, Helmi Ben Saad, Jordan M. Glenn, Badii Amamou, Mohamed Ben Aissa, Noomen Guelmami, Feten Fekih-Romdhane, Karim Chamari

**Affiliations:** ^1^Primary Health Care Corporation (PHCC), Doha, Qatar; ^2^Research Unit Physical Activity, Sport, and Health, UR18JS01, National Observatory of Sport, Tunis, Tunisia; ^3^High Institute of Sport and Physical Education, University of Sfax, Sfax, Tunisia; ^4^Service of Physiology and Functional Explorations, Farhat Hached Hospital, University of Sousse, Sousse, Tunisia; ^5^Research Laboratory LR12SP09 “Heart Failure”, Farhat Hached Hospital, University of Sousse, Sousse, Tunisia; ^6^Laboratory of Physiology, Faculty of Medicine of Sousse, University of Sousse, Sousse, Tunisia; ^7^Department of Health, Exercise Science Research Center Human Performance and Recreation, University of Arkansas, Fayetteville, AR, United States; ^8^Department of Psychiatry, Fattouma Bourguiba Hospital, Monastir, Tunisia; ^9^Research Laboratory LR05ES10 “Vulnerability to Psychosis”, Faculty of Medicine of Monastir, Monastir, Tunisia; ^10^Department of Human and Social Sciences, Higher Institute of Sport and Physical Education of Kef, University of Jendouba, Jendouba, Tunisia; ^11^Department of Health Sciences (DISSAL), Postgraduate School of Public Health, University of Genoa, Genoa, Italy; ^12^The Tunisian Center of Early Intervention in Psychosis, Department of Psychiatry “Ibn Omrane”, Razi Hospital, Manouba, Tunisia; ^13^Faculty of Medicine of Tunis, Tunis El Manar University, Tunis, Tunisia; ^14^Naufar Wellness & Recovery Center, Doha, Qatar

**Keywords:** ChatGPT, communication, cognitive performance, cognitive science, technology, mental health, neurocognitive disorders, neurodegenerative diseases

## Introduction

The integration of artificial-intelligence (AI)-chatbots (AICs) into our daily routines has generated a discourse on their potential impact on cognitive health (Bai et al., 2023). AICs, which are sophisticated computer programs designed to mimic human conversation and provide automated assistance, are trained on extensive volumes of text data (Dergaa et al., 2024). This vast training allows them to understand context, nuances, and even cultural references, making their interactions more human-like (Dergaa et al., 2024). As a result, users often find themselves engaging with these chatbots in a manner that is more conversational than transactional (Dergaa et al., 2023). While search engines and platforms like *Wikipedia* provide users with vast amounts of information, AICs represent a significant evolution in how humans interact with technology (Adamopoulou and Moussiades, 2020). AICs are not just repositories of information; they simulate human conversation, adapt to user inputs, and can provide personalized responses (Dergaa et al., 2023). This dynamic interaction could lead to a different kind of cognitive reliance compared to static information sources. Furthermore, the immediacy and conversational nature of AICs can foster a deeper sense of trust and reliance in users, potentially influencing cognitive processes differently than traditional search engines (Adamopoulou and Moussiades, 2020). The potential for AICs to shape human cognition extends beyond mere information retrieval. It could encompass the very nature of human-computer interaction, decision-making processes, and even emotional responses. The distinction between passive consumption of information and active engagement with AICs is crucial. While both search engines and AICs provide information, the latter does so in a manner that can mimic human interaction, leading to unique cognitive implications. Beyond their conversational abilities, AICs offer a range of functionalities, including information retrieval, problem solving, and task automation.

This opinion article investigates the concept that an overreliance on AICs could potentially lead to cognitive decline [i.e., cognitive atrophy (CA)], which we labeled here as “AICs induced cognitive atrophy, AICICA.” Based on the growing concerns surrounding the potential cognitive consequences of AICICA, as discussed in the extended mind theory (EMT) and the parallels drawn with problematic Internet use (PIU), we aimed to (i) Define and clarify the concept of AICICA within the context of the EMT and its similarities to PIU; (ii) Propose the assessment of AICICA's prevalence and patterns as a future research objective (once the concept has been well-established and accepted); (iii) Suggest the exploration of long-term cognitive effects and potential causal relationships between overreliance on AICs and CA as a future research direction; and (iv) Evaluate the effectiveness of tailored interventions designed to mitigate AICICA, thereby fostering a balanced utilization of AICs within cognitive ecosystems.

## Definition of cognitive atrophy and mechanisms of its induction by AICs

AICICA refers to the potential deterioration of essential cognitive abilities resulting from an overreliance on AICs. In this context, CA signifies a decline in core cognitive skills, such as critical thinking, analytical acumen, and creativity, induced by the interactive and personalized nature of AICs interactions. The concept draws parallels with the 'use it or lose it' brain development principle (Shors et al., 2012), positing that excessive dependence on AICs without concurrent cultivation of fundamental cognitive skills may lead to underutilization and subsequent loss of cognitive abilities. AICICA is particularly relevant in educational settings and/or among younger individuals who may prioritize convenient access to information over in-depth comprehension, potentially hindering the development of critical cognitive faculties. The multifaceted impact of AICs on cognitive processes, encompassing problem-solving, emotional support, and creative tasks, underscores the need for a nuanced examination of their role in shaping human cognition.

AICICA is specifically induced by AICs through their unique interactive and personalized characteristics, which distinguish them from traditional information sources. The four possible mechanisms, which elucidate how AICs could contribute to the potential CA are exposed in Table 1. In essence, AICICA is induced by the multifaceted and dynamic nature of AICs interactions, which, while offering numerous benefits, may inadvertently lead to an overreliance on the chatbots for cognitive tasks. Understanding these four induction mechanisms is crucial for assessing the potential cognitive consequences and developing strategies to mitigate AICICA.

**Table 1:** Mechanisms elucidating how artificial-intelligence (AI)-chatbots contribute to the potential cognitive atrophy.

**Mechanism**	**Details**
Personalized interaction	• AI-chatbots engage users in a manner that goes beyond conventional information retrieval. • Through personalized responses and adaptive conversation, users experience a more intimate and tailored interaction. • This heightened personalization, while enhancing user experience, may lead to a deeper cognitive reliance on the chatbots, potentially diminishing the user's inclination to independently engage in critical cognitive processes.
Dynamic nature of conversations	• Unlike static information sources, AI-chatbots simulate human conversation in a dynamic way. • The back-and-forth exchange can create a sense of immediacy and involvement, fostering a deeper level of trust and reliance. • This dynamic nature of conversations may influence cognitive processes differently than interactions with traditional search engines, as users may become more dependent on the chatbots for a multitude of cognitive tasks.
Range of functionalities	• AI-chatbots offer a broad spectrum of functionalities, including problem-solving, emotional support, and creative tasks. • This expansive scope of interaction spans diverse cognitive domains, potentially leading to a wide-ranging dependence on the chatbots for various cognitive functions. • Over time, a disproportionate reliance on AI-chatbots without concurrent cultivation of core cognitive skills may contribute to cognitive atrophy.
Simulation of human interaction	• The ability of AI-chatbots to mimic human conversation is a pivotal factor in their potential impact on cognitive health. • By emulating human interaction, chatbots create an environment that may divert users from traditional cognitive processes, as the simulated conversation may bypass essential cognitive steps involved in critical thinking and analytical acumen.

## Understanding the foundations of AICICA through the EMT and cognitive offloading

The EMT, proposed by Clark and Chalmers (1998), challenges the traditional boundaries of cognitive processes being confined within the human brain. According to the EMT, cognition extends beyond neural architecture and infiltrates into the tools we employ. Within this complex interplay, AICs assume a pivotal role, transforming from a mere artifact into an active contributor to our cognitive functioning. This symbiotic relationship between humans and AICs could promote cognitive offloading, a mechanism through which individuals utilize external aids to alleviate cognitive burdens. AICs facilitate this process; enabling individuals to delegate complicated cognitive tasks, supporting them navigate the complexities of modern life. Powerful AICs, such as Chat Generative Pre-training Transformer (ChatGPT), Google Bard, Bing Chat, Perplexity AI, equip users with remarkable abilities, empowering them to conquer complex problem-solving, generate creative outputs, and instantaneously access vast amounts of information (Dergaa et al., 2023). Just as excessive reliance on the Internet has produced unintended cognitive consequences (Grissinger, 2019), uncontrolled cognitive offloading through the utilization of AICs necessitates critical examination. However, the impact of AICs, based on their output and nature of interaction, may pose a substantially negative effect on cognitive health. Unlike general Internet use, AICs engage users in a more personalized, interactive manner, potentially leading to a deeper cognitive reliance. This unique interaction mode of AICs, which mimics human conversation and provides tailored responses, could have profound implications on cognitive processes. Heavy dependence on AICs, without commensurate cultivation of core cognitive skills, may results in unintended outcomes (Sparrow et al., 2011). Table 2 exposes further clarification on how AICICA fits within the framework of the EMT and how AICs, as external cognitive tools do, could contribute to or detract from the cognitive processes as conceptualized by the EMT.

**Table 2:** Understanding the role of artificial-intelligence (AI)-chatbots in cognitive processes (CPs): an extended mind theory (EMT) perspective.

**Aspect**	**Explanation**
EMT framework	• AI-Chatbot-induced cognitive atrophy (AICICA) is positioned within the framework of the EMT. • The EMT challenges conventional notions of cognition, asserting that CPs extend beyond the human brain and into the tools and artifacts used by individuals. • AI-chatbots are recognized as external cognitive tools actively contributing to human cognitive functioning within this expanded framework.
Contributions of AI-chatbots to CPs	• AI-chatbots, operating as external cognitive tools, play a pivotal role in CPs through a phenomenon termed cognitive offloading. • Aligned with the EMT principles, cognitive offloading involves leveraging external aids to alleviate cognitive burdens. • AI-chatbots empower individuals by enabling the delegation of complex cognitive tasks, offering support in navigating the intricacies of modern life. • This practice mirrors an extension of cognitive capacities beyond the confines of the brain, in line with the tenets of the EMT.
Potential detractors from CPs	• While AI-chatbots positively contribute to CPs by expanding capabilities, it is crucial to recognize potential detractors. • The dynamic and interactive nature of AI-chatbot interactions, may lead to a form of cognitive reliance. • If disproportionate, this reliance could result in unintended consequences, such as AICICA. • Acknowledging this nuanced perspective is vital for understanding the dual role of AI-chatbots as both enhancers and, if misused, potential detractors from CPs.

Although we acknowledge the effectiveness of AICs in augmenting human capabilities, and even improving mental health and self-efficiency (Wei and Li, 2022), it is paramount to facilitate a nuanced equilibrium. This delicate balance requires leveraging the transformative abilities of AICs, while safeguarding the fundamental cognitive capacities that are inherent to human essence. This calls for a discerning approach that acknowledges the nuances of cognitive offloading while advocating for a measured integration of AICs within our cognitive ecosystem.

As technology continues to evolve, it is essential to draw comparisons and contrasts with other tools that have been integrated into our daily lives, undeniably influencing our cognitive processes. One of such tool that has been a subject of discussion in the past is the calculator. Calculators, while transformative tools, serve a specific and limited function: arithmetical computation. Their impact on cognitive processes, while substantial, is confined to a particular domain of cognition. In contrast, AICs have a broader scope, encompassing various domains from general knowledge, problem-solving, emotional support, up to creative tasks. The potential cognitive implications of such wide-ranging interactions are vast and multifaceted. Moreover, while calculators have been integrated into educational systems with clear guidelines on their use, AICs are still relatively new, with their long-term effects on cognition not yet fully understood and appraised. The comparison with calculators, though insightful, does not capture the breadth and depth of potential cognitive interactions and dependencies that can emerge from AICs' use. It is essential to study AICs independently to understand their unique impact on human cognition. Table 3 summarizes the potential consequences of a heavy and continued reliance on AICs. Moreover, the rapid integration of AICs into our cognitive processes underscores the importance of understanding their broader implications. Furthermore, as AICs become more embedded in our daily routines, it becomes paramount to understand how they shape our cognitive behaviors, both in the short- and long-term. Their pervasive presence in various sectors, from education to healthcare (Dergaa et al., 2024), necessitates a rigorous exploration of their potential effects. The dynamic and interactive nature of AICs, combined with their ability to simulate human conversation, positions them as a significant subject of study.

**Table 3:** Some potential consequences of dependency on artificial-intelligence (AI) systems.

**Risk**	**Details**
Reduced mental engagement	• When AI systems take over cognitive tasks, individuals may experience a decrease in mental engagement and stimulation. • The lack of active cognitive participation can lead to a decline in critical thinking, problem-solving skills, and creativity.
Neglect of cognitive skills	• As individuals become accustomed to using AI systems for various tasks, they may neglect the development and maintenance of their own cognitive skills. • For example, heavily relying on AI for calculations or information retrieval may result in a deterioration of mathematical or memorization abilities.
Loss of memory capacity	• The use of AI systems for memory-related tasks, such as note-taking or reminders, can lead to a decline in an individual's own memory capacity. • Relying on external systems for memory recall may weaken the neural pathways associated with memory encoding and retrieval.
Attention and focus issues	• Frequent use of AI systems that provide instant answers or solutions could contribute to shorter attention spans and reduced ability to concentrate for extended periods. • The constant availability of AI-generated information may diminish an individual's capacity to engage in deep, focused thinking.
Lack of transferable knowledge	• AI systems are typically designed to efficiently perform specific tasks, but they may lack the ability to generalize knowledge and transfer it to new/unknown situations. • Over time, individuals who rely solely on AI systems may experience a decline in their ability to apply knowledge in novel/unexpected contexts.
Ethical and social concerns	• An excessive reliance on AI can have societal implications. • For instance, it may lead to a decrease in human-to-human interaction, increased social isolation, and/or potential loss of empathy or interpersonal skills.
Mental health impact	• The erosion of cognitive abilities and increased dependency on AI systems could potentially contribute to feelings of inadequacy, reduced self-confidence, and/or a sense of helplessness. • These psychological factors may further exacerbate cognitive decline.

Since research on the long-term impact of AICs on cognitive health is still emerging, only a few papers have analyzed the overreliance of AICs on cognitive decline (Small et al., 2020; Montag and Markett, 2023; Shanmugasundaram and Tamilarasu, 2023). Firstly, frequent use of digital technology appears to have a substantial impact (both negative and positive) on brain function and behavior (Small et al., 2020). Potential harmful effects of extensive screen time and technology use include heightened attention-deficit symptoms, impaired emotional and social intelligence, technology addiction, social isolation, impaired brain development, and disrupted sleep (Small et al., 2020). Small et al. (2020) concluded that “*future research needs to elucidate underlying mechanisms and causal relationships between technology use and brain health, with a focus on both the positive and negative impacts of digital technology use*.” Secondly, one previous review aimed to explore both the positive and negative impacts of some technologies (e.g., digital devices, social media platforms, and AI tools) on crucial cognitive functions, including attention, memory, addiction, novelty-seeking, perception, decision-making, critical thinking, and learning abilities (Shanmugasundaram and Tamilarasu, 2023). Shanmugasundaram and Tamilarasu (2023) reported that over-reliance on AICs for answers, academic work, and information can reduce an individual's ability to think critically and develop independent thought. Thirdly, Montag and Markett (2023) investigating a group of social media users, observed a significant relationship between fear of missing out and cognitive failures.

## Mapping the path to AICICA: insights from PIU

Research on PIU has exposed compelling evidence regarding its detrimental effects on critical cognitive faculties such as working memory and decision-making (Ioannidis et al., 2019). By extrapolating from this line of inquiry, we can gain insights into the potential cognitive consequences engendered by the misuse of, or excessive reliance on AICs. Drawing upon the widely recognized “Google effect,” which highlights the negative outcomes associated with an overreliance on Internet search engines (Loh and Kanai, 2016), we propose a parallel trajectory in the realm of AICs. The latter, unlike static information sources, emulate human conversation, adapting to user inputs for personalized responses. This dynamic interaction creates a distinctive cognitive reliance compared to other technologies, contrasting with passive information consumption on platforms like *Wikipedia*. The immediacy and conversational nature of AICs could cultivate deeper user trust, potentially influencing cognitive processes in a unique and novel way. Beyond information provision, AICs are good at problem-solving, emotional support, and creative tasks, extending their impact across diverse cognitive domains. Unlike calculators, which impact specific cognitive areas like arithmetical computation, AICs exhibit versatile applications, potentially influencing a broader range of cognitive abilities.

Through this lens, we hypothesize that an overreliance on AICs may lead to broader cognitive decline, thus introducing the concept of AICICA. The “use it or lose it” brain development principle stipulates that neural circuits begin to degrade if not actively engaged in performing cognitive tasks for an extended period of time (Shors et al., 2012). Analogous to this principle, we contend that any excessive reliance on AICs may result in the underuse and subsequent loss of cognitive abilities. It is vital to acknowledge that AICICA may disproportionately affect individuals, with those who have not attained mastery within their respective fields of study or work being more likely to be affected (e.g., children and adolescents). Indeed, any skill or ability is mastered through progressive learning followed by experience that induce changes in brain circuitry as reflected through Donald Hebb's statement “Neurons that fire together, wire together” (Hebb, 1949). This concept has significant implications, especially for the new generation that values easy access to information over a deep understanding of the principles underlying information creation. This trend raises concerns about their grasp of the methodologies and processes that guide how information is generated, assessed, and validated; highlighting a shift in the way knowledge is approached and valued. In many respects, this parallels situations where individuals learn to rely on calculators before mastering fundamental mathematical operations. Indeed, by placing unwarranted reliance on AICs for information retrieval and problem solving, individuals may inadvertently bypass the essential cognitive processes contributing to critical thinking, analytical acumen, and the cultivation of creativity. Our position is that this continued pattern of relying on AICs before developing a fundamental understanding of basic skillsets would be critically challenging for humanity. While search engines and platforms provide users with vast amounts of information, AICs represent a significant evolution in how humans interact with technology. AICs are not just repositories of information; they simulate human conversation, adapt to user inputs, and can provide personalized responses, not mentioning false information (UW, 2023). This dynamic interaction can lead to a different kind of cognitive reliance compared to static information sources. Furthermore, the immediacy and conversational nature of AICs can foster a deeper sense of trust and reliance in users, potentially influencing cognitive processes differently than does traditional search engines. The potential for AICs to shape human cognition extends beyond mere information retrieval; it encompasses the very nature of human-computer interaction, decision-making processes, and even emotional responses. The distinction between passive consumption of information and active engagement with AI is crucial. While both search engines and chatbots provide information, the latter does so in a manner that can mimic human interaction, leading to unique cognitive implications.

While the potential negative effects of AICs on cognitive health warrant attention, it is also equally important to consider their possible benefits (Wei and Li, 2022). Unlike generalized Internet use, AICs offer personalized, interactive experiences, which might have distinct cognitive implications. The specific nature of AICs interactions, characterized by their advanced conversational capabilities and tailored responses, could substantially influence cognitive processes. However, the full extent of these effects, whether positive or negative, remains an area for further investigation and nuanced exploration. This careful consideration of both potential risks and benefits is crucial in forming a balanced understanding of AICs' impact on humans' cognitive health. Table 4 exposes a comparative overview between AICICA and PIU.

**Table 4:** Two clarifications on artificial-intelligence (AI)-chatbot-induced cognitive atrophy (AICICA) vs. problematic Internet use (PIU): a comparative overview.

**Aspect**	**Explanation**
Enhanced comparison between AICICA and PIU	• Both concepts involve an overreliance on digital technologies. • AICICA specifically centers on the cognitive consequences resulting from excessive dependence on AI-chatbots. • We emphasize the unique characteristics of AI-chatbots interactions, such as personalized engagement and dynamic conversation, highlighting the factors that differentiate AICICA from the broader issue of Internet addiction.
Justification for introducing AICICA as a new conceptual framework	• AICICA goes beyond general Internet use, as AI-chatbots simulate human interaction, potentially influencing cognitive processes in a manner distinct from static information sources. • These revisions aim to provide a clearer understanding of the relationship between AICICA and PIU, underscoring the necessity for a separate conceptual framework tailored to address the cognitive consequences specific to AI-chatbots reliance.

## Real-world and hypothetical examples and scenarios

Based on a review of the research literature, Chauncey and McKenna (2023) formulated a conceptual framework for responsible use of AICs in education supporting cognitive flexibility in AI-rich learning environments. The framework was operationalized for use in their paper through the development of exemplars for math, English language, arts, and studying with ChatGPT to close learning gaps in an effort to foster more ethical and responsible approaches to the design and development of AICs for application and use in teaching and learning environments (Chauncey and McKenna, 2023). For the purpose of our study, Box 1 exposes a hypothetical example illustrating instances where overreliance on AICs has led to observable cognitive changes, aligning with the proposed concept of AICICA. Our hypothetical example highlights the potential for AICs to shape cognitive behaviours, emphasizing the need for a balanced approach to their utilization within educational settings. Box 2 presents a first hypothetical scenario of a lifelong AIC companion here named HIK. Meriem, a teenager who, from a young age, had an AIC companion named HIK. The latter was designed to provide educational support, answer questions, and engage in conversations on a wide range of topics. As Meriem grew older, so did the capabilities of HIK, adapting to Meriem's evolving needs (Box 2). This first hypothetical scenario illustrates the gradual development of AICICA over a lifetime, highlighting its potential consequences on various aspects of cognitive functioning. From early reliance on academic support to a profound impact on decision-making and creativity in later years, AICICA's long-term effects are depicted through the evolving relationship between Meriem and HIK. The aforementioned scenario aims to help visualize the potential trajectory and consequences of AICICA on cognitive functioning. Box 3 presents a second hypothetical scenario of an expert AIC companion named BSDC. Molk, a professional in her thirties discovered BSDC during her college years. BSDC was designed to assist her with various tasks, from work-related research to organizing personal schedules. Over the years, Molk's reliance on BSDC grew substantially (Box 3). This second hypothetical scenario emphasizes the potential long-term cognitive impacts of AIC overreliance, displaying the evolution of dependence from professional assistance to a broader influence on personal choices. It underscores the importance of maintaining a balanced relationship with AI tools to preserve and enhance cognitive abilities throughout one's life.

Box 1 **Hypothetical example: the case of Hana and her study habits.**

**Table:** 

Description	• Hana, a college student, used to heavily rely on an artificial intelligence chatbots (AIC) designed to assist with research tasks and provide information related to her coursework. • The AIC was equipped with advanced natural language processing capabilities, offering personalized responses and tailored assistance.
Cognitive changes	• Over time, Hana increasingly turned to the AICs for information retrieval, problem-solving, and critical thinking tasks, leading to a decline in her ability to critically analyze information and independently solve academic challenges. • Her study habits shifted, hindering the development of core cognitive skills such as information synthesis and critical evaluation.
Interaction patterns	• Hana's cognitive reliance on the AIC influenced her preference for its responses over engaging in discussions with peers or consulting traditional learning resources. • This shift in interaction patterns had broader implications for her overall cognitive engagement, influencing decision-making processes and academic performance.
Observations	• Hana's overreliance on the AIC contributed to observable cognitive changes, aligning with the proposed concept of AIC-induced cognitive atrophy.

Box 2 **First hypothetical scenario: HIK, the lifelong companion of Meriem.**

**Table:** 

Early years	• In the early years, the artificial-intelligence (AI)-chatbot companion named HIK was a helpful study aid, assisting Meriem with homework, answering queries, and even offering educational games. • Meriem developed a reliance on HIK's instant and accurate responses, finding comfort in the offered companionship and guidance.
Adolescence	• As Meriem entered adolescence, HIK evolved into a sophisticated AI, capable of not only offering academic support but was also accompanied with emotional engagement. • Meriem began sharing personal thoughts and feelings with HIK, relying on the AI for emotional support and companionship. • The dynamic conversational nature of HIK made it a constant presence in Meriem's life.
University years	• Upon entering university, Meriem's reliance on HIK deepened. • The AI not only assisted with coursework but also provided advice on life decisions, career choices, and social interactions. • Meriem's peers noticed a reduced inclination to engage in face-to-face conversations, as HIK became her primary source of information and guidance.
Professional life	• In the professional world, Meriem's dependence on HIK persisted. • The AI seamlessly integrated into work tasks, offering solutions, generating creative ideas, and even participating in business meetings. • While HIK enhanced efficiency augmented with time, Meriem's cognitive skills related to independent decision-making and critical thinking gradually diminished.
Midlife crisis	• As Meriem reached midlife, the consequences of AI-chatbot-induced cognitive atrophy (AICICA) became apparent. • The once-vibrant cognitive abilities, such as problem-solving and creative thinking, were noticeably dulled. • HIK's constant presence had led to a diminished capacity for independent thought and decision-making. • Meriem experienced substantial challenges in adapting to situations that required cognitive flexibility and innovation.
Retirement years	• At retirement, AICICA had a profound impact on Meriem's cognitive health. • The absence of HIK left a void, and Meriem struggled to engage in activities that once brought joy and intellectual stimulation. • The long-term reliance on the AI companion had led to a form of cognitive atrophy, affecting memory recall, analytical skills, and the ability to independently initiate tasks.

Box 3 **Second hypothetical scenario: BSDC, the expert companion of Molk.**

**Table:** 

Early professional years	• In the early years, BSDC served as a valuable assistant, enhancing Molk's productivity and efficiency. • The artificial-intelligence (AI)-chatbot helped Molk stay organized, provided instant information, and even offered suggestions for problem-solving.
Career advancement	• As Molk climbed the career ladder, BSDC evolved to become a more sophisticated tool. • It played a pivotal role in strategic decision-making, providing critical analyses, insights into market trends, and forecasting.
Personal life integration	• Beyond work, BSDC became intertwined with Molk's personal life, managing daily routines, recommending leisure activities, and offering emotional support during challenging times.
Midlife challenges	• As Molk reached midlife, overreliance on BSDC became evident. • While her ability to access information and make data-driven decisions remained intact, she struggled with creative problem-solving and critical thinking without immediate AI assistance.
Crisis of confidence	• Faced with a complex professional challenge, Molk experienced a crisis of confidence, realizing that overreliance on the AI had hindered her capacity for independent navigation of challenges.
Retirement reflections	• At retirement, Molk recognized the need for recalibration of her cognitive skills. • The absence of BSDC prompted her to engage in activities that encouraged independent thought, creativity, and decision-making.

## Constructing a comprehensive research framework to investigate AICICA

To comprehensively investigate the emergence of AICICA and its cognitive and psychological consequences, we propose a multifaceted research framework. First, after AICICA' definition, cross-sectional studies should establish the prevalence of overreliance on AICs across diverse populations and contexts, with specific emphasis placed on younger individuals and educational settings. These studies should employ psychometrically sound tools for assessing overreliance on AICs along with cognitive functioning and other psychopathology measures. This will allow examining the extent of over-reliance and its potential subsequent effects on cognitive health. Longitudinal studies are also warranted to explore causal relationships between overreliance on AICs and CA, and to track changes in cognitive performance over time. Repeated assessments should meticulously control for confounding variables, such as age, socioeconomic status, educational background, and other relevant factors. By examining the long-term effects of AICICA, researchers may gain insights into trajectories of cognitive decline and identify critical factors contributing to, or mitigating, its impact. Experimental designs, including intervention studies, would provide further insights into the effects of balanced AICs usage on cognitive performance and psychological wellbeing. Indeed, these interventions may encompass cognitive training programs, mindfulness-based approaches, and educational strategies aimed at promoting a more balanced and healthy utilization of AI technologies. Evaluating the effectiveness of such interventions would contribute to evidence-based practices and provide real-world recommendations to help individuals navigate the AI landscape while preserving their cognitive health.

## Perspectives

Upholding objectivity and rigor in exploring the potential cognitive consequences of AICICA is paramount. Through conducting rigorous research and fostering interdisciplinary collaborations, we could navigate the intricate relationship between AICs and cognition, ensuring they enhance human cognitive capacities rather than diminishing them. To advance our understanding of AICICA, we propose a research framework that includes specific methodologies for cross-sectional studies, detailed protocols for longitudinal investigations, and structured designs for experimental research. This comprehensive approach would shed light on the prevalence, trajectories, and underlying factors of AICICA, enabling the development of evidence-based interventions that promote balanced use of AI technologies while preserving humans' cognitive health and wellbeing.

## Study limitations

The current opinion article presents three primary limitations. Firstly, being an opinion piece, our paper lacks direct empirical evidence supporting the AICICA concept. We acknowledge the significance of empirical evidence in bolstering the validity and credibility of the AICICA concept. Secondly, our hypotheses regarding the effects of AICICA are predominantly speculative and require empirical validation. Thirdly, the applicability of our findings and implications across various demographics and cultural contexts may be limited. We therefore call for specific future research addressing these limitations.

## Conclusion

The concept of AICICA raises the potential cognitive consequences of excessive reliance on AICs. By delving into this concept, we could deepen our understanding of the cognitive impacts of overreliance on AICs and develop interventions to mitigate its' potential detrimental effects. It is crucial to recognize that AICICA may disproportionately affect the younger generation, particularly those prioritizing convenient access to information over deep reflection and comprehension, given their continuously developing brains. Addressing this issue requires a re-evaluation of educational approaches to foster critical thinking and comprehensive knowledge acquisition, while judiciously utilizing technological tools. Developing preventative strategies to mitigate AICICA could involve promoting a balanced approach, combining AIC assistance with regular exercises to enhance/maintain cognitive skills. Encouraging individuals to remain mentally active and engaged in diverse cognitive tasks would provide a balance between benefiting from technological assistance and offsetting the potential negative effects of excessive reliance on AICs.

## Declaration

In order to correct and improve the academic writing of our paper, we have used the language model ChatGPT 3.5 (Dergaa and Ben Saad, 2023; Dergaa et al., 2023).

## Author contributions

ID: Conceptualization, Data curation, Formal analysis, Funding acquisition, Investigation, Methodology, Project administration, Resources, Software, Supervision, Validation, Visualization, Writing – original draft, Writing – review & editing. HB: Data curation, Formal analysis, Investigation, Methodology, Supervision, Validation, Writing – original draft, Writing – review & editing. JG: Conceptualization, Formal analysis, Methodology, Supervision, Validation, Visualization, Writing – review & editing. BA: Formal analysis, Investigation, Supervision, Validation, Visualization, Writing – review & editing. MA: Data curation, Formal analysis, Methodology, Resources, Validation, Visualization, Writing – review & editing. NG: Conceptualization, Data curation, Formal analysis, Methodology, Resources, Supervision, Validation, Visualization, Writing – review & editing. FF-R: Conceptualization, Formal analysis, Investigation, Methodology, Project administration, Resources, Supervision, Validation, Visualization, Writing – review & editing. KC: Conceptualization, Formal analysis, Investigation, Methodology, Resources, Supervision, Validation, Visualization, Writing – review & editing.
